# Natural History and Prognostic Factors at First Relapse in Multiple Myeloma

**DOI:** 10.3390/cancers12071759

**Published:** 2020-07-02

**Authors:** Chen Wang, Cinnie Yentia Soekojo, Sanjay de Mel, Melissa Ooi, Yunxin Chen, Allan Zhi Kai Goh, Chandramouli Nagarajan, Wee Joo Chng

**Affiliations:** 1 Department of Haematology-Oncology, National University Cancer Institute, National University Health System, Singapore 119074, Singapore; chen.wang744p@gmail.com (C.W.); cinnie_yentia_soekojo@nuhs.edu.sg (C.Y.S.); sanjay_widanalage@nuhs.edu.sg (S.d.M.); melissa_ooi@nuhs.edu.sg (M.O.); 2 Department of Haematology, Singapore General Hospital, Singapore 169608, Singapore; chen.yunxin@singhealth.com.sg (Y.C.); allan.goh.z.k@sgh.com.sg (A.Z.K.G.); chandramouli.nagarajan@singhealth.com.sg (C.N.); 3 Department of Medicine, Yong Loo Lin School of Medicine and National University of Singapore, Singapore 119228, Singapore; 4 Cancer Science Institute of Singapore and National University of Singapore, Singapore 117599, Singapore

**Keywords:** myeloma, relapse, survival

## Abstract

The prognosis of multiple myeloma has considerably improved due to the introduction of novel agents in the upfront setting. However, the great majority of patients ultimately relapse, and choosing a salvage treatment at first relapse remains challenging. The natural history of first relapsed disease in the current era is also not well described. We retrospectively studied 300 patients with first relapsed myeloma seen between 2004 and 2019 from two institutes in Singapore. The median duration from diagnosis to first relapse was 22.7 months (1.1–97.0 months). Most patients received novel agent-based induction therapy, and 41.3% underwent autologous stem cell transplant. A very good partial response (VGPR) or better was achieved in 48.6%. Regarding first relapse, 50.5% were symptomatic and 19.0% received newer agent-containing regimens. Nearly a third of patients (31.7%) had a VGPR or better response. The median progression free and overall survival from first relapse was 12.0 and 44.8 months, respectively. Based on a randomized sample splitting, we first identified non-hyperdiploid karyotype at diagnosis, clinical relapse, and treatment sequence as impacting survival independently from a testing cohort, and we then further demonstrated their significance in a validation cohort. This study provides a real-world picture of first relapsed myeloma and highlights the prognostic importance of the treatment sequence.

## 1. Introduction

Multiple myeloma (MM) is an incurable malignant plasma cell disorder [1]. Treatment options for MM have expanded with the introduction of several classes of novel agents, which improve the prognosis considerably [2,3]. However, almost all patients will eventually relapse and require further treatment. Determining the optimal salvage regimen at first relapse remains challenging, as numerous factors need to be considered, including prior treatment used as well as the depth and duration of response [4,5]. Unfortunately, the further response is usually inferior to initial treatment with progressively shorter duration of remission [6]. 

Therapeutic decisions for first relapse patients should be approached with the use of results from randomized trials in the relapsed setting, which have shown the superiority of triplet (triple combination) in terms of response and progression free survival [7,8,9,10,11,12]. Even so, there are still multiple possibilities and the decision is always influenced by a patient’s functional status and comorbidities. In addition, cost is an unavoidable concern in view of the huge economic disparities, especially for triplet regimens with two novel agents [13]. Therefore, the decision is not easily justified by using trial data alone, but by gaining more information from real-world practice, which is adapted according to personal resources, healthcare infrastructure, and even local cultures [14].

Rising incidence of MM in Asia has been reported, and there are approximately 100 newly diagnosed cases per year in Singapore [15,16]. Given the drastic changes of treatment approach and increasing access to novel agents in our daily practice, it is important to study the natural history of first relapsed MM in a real-world setting. Herein, we described the real-world assessment of patients with first relapsed MM, with a focus on identifying prognostic factors for post-relapse survival. 

## 2. Methods

### 2.1. Patients

Patients with first relapsed MM seen at National University Hospital and Singapore General Hospital, Singapore between April 2004 and May 2019 were included in the current study. Disease-associated parameters, including baseline assessment at diagnosis, initial treatment and response, pattern of first relapse, re-assessment at first relapse, salvage treatment, and response, were obtained from medical records. Interphase fluorescence in-situ hybridization (FISH) was performed as described in Supplementary Method. High-risk features, detected by locus-specific probes, include FGFR3/IGH [t(4;14)(p16;q32)], IGH/MAF [t(14;16)(q32;q23)], and TP53 [del(17p13)]. Institutional Review Boards approved the study (National Healthcare Group Domain Specific Review Board reference numbers 2007/00173 and 2012/00058), and the use of patient medical records was conducted in accordance with the principles of the Declaration of Helsinki.

The primary objective of the current study was to describe survival after first relapse and identify prognostic factors. Overall survival (OS) and progression free survival (PFS) were defined as the time from start of salvage therapy for first relapse to death or earlier date of further relapse/death, respectively. PFS is the primary endpoint, as it is more indicative of the salvage regimen’s efficacy in controlling relapse. Treatments were categorized as conventional therapy (i.e., alkylator-based, palliative steroid, and best supportive care), novel agent-based regimens (i.e., bortezomib, thalidomide, and lenalidomide), and newer agent-containing regimens (i.e., daratumumab, carfilzomib, pomalidomide, ixazomib, and dinaciclib, a cyclin-dependent kinases (CDK) inhibitor that has been reported previously [17]). Treatment response, relapse, and progression were defined according to International Myeloma Working Group consensus criteria. In brief, clinical relapse requires direct indicators of increasing disease (e.g., plasmacytoma) and/or end organ dysfunction (i.e., hypercalcemia, renal insufficiency, anemia, and lytic bone lesions) [18]. 

### 2.2. Statistical Analyses

Data analysis was performed with SPSS version 22.0 (SPSS, Inc., Chicago, IL, USA). The whole-patient cohort (n = 300) was randomly assigned to a testing cohort (n = 150) and an independent validation cohort (n = 150) based on a split-sample method [19]. Differences in categorical variables were compared with the χ^2^-test (using Fisher’s exact test when appropriate) and with the Mann–Whitney test for continuous variables.

Survival curves were plotted with the Kaplan–Meier method and compared with a log-rank test. The Cox proportional hazards model was used to assess the association of relevant clinical variables with survival. Only variables with certain significance (*p* < 0.10) in univariate analysis were included in the multivariate model. In terms of treatment sequence analysis, patients with different treatment sequences but similar survival trends were combined to generate subgroups. All data were considered statistically significant at *p* < 0.05.

## 3. Results

### 3.1. Patients’ Characteristics

The study included 300 first relapsed MM patients, equally split into the testing and validation cohorts. Overall, 166 patients (55.3%) were male. The median age at relapse was 66 years (range 36–94) with a median duration from diagnosis to first relapse of 22.7 months (range 1.1–97.0). Patients’ characteristics are shown in Table 1, and clinical features were comparable in the two split cohorts. The baseline international staging system (ISS) distribution, cytogenetics (metaphase karyotyping), and fluorescence in-situ hybridization (FISH) findings were typical of newly diagnosed MM. Before first relapse, most patients (94.7%) received a novel agent-based regimen as the initial treatment (Table S1) and 124 (41.3%) underwent upfront autologous stem cell transplant (ASCT). Best responses were categorized in 290 patients with complete data, and 48.6% achieved a very good partial response (VGPR) or better. 

Clinical relapse occurred in 145 patients (50.5%), and the remaining showed biochemical relapse. New development of extramedullary plasmacytoma was documented in 43 patients (21.3%). Cytogenetics and FISH were not routinely examined at relapse. Newer agent-containing regimens were given in 57 patients (19.0%) (Table S2), and only 17 (5.7%) received a second ASCT. However, less than a third of patients (31.7%) reached a VGPR or better (Table S3). The rates of response varied according to salvage regimens. A VGPR or better was achieved in 45.6% of patients using a newer agent-containing regimen, as compared with 27.4% and 12.9% in those who received a novel agent-based regimen and conventional therapy, respectively (*p* < 0.001) (Figure S1). 

### 3.2. Treatment Sequences by Comparing Initial and Salvage Regimens

Comparing the backbone agents of initial and salvage regimens showed several typical treatment sequences in the current study, including conventional therapy as salvage regardless of the initial regimen, same novel agent, generation escalation of the same class of novel agent (e.g., bortezomib escalated to carfilzomib), class switch of the novel agent (e.g., proteasome inhibitor switched to immunomodulatory drug and vice versa), daratumumab as salvage, and others (e.g., adding on a novel agent) (Table S4). To determine the prognostic impact of the treatment sequence, patients were categorized based on their survival trends, which generated three different prognostic subgroups, namely de-escalation (conventional chemotherapy or best supportive care as salvage), backbone change (class switch or daratumumab as salvage), and others (median PFS 4.6 vs. 20.7 vs. 12.0 months, *p* < 0.001) (Figure S2). 

In addition, PFS was also analyzed based on the class of novel agent received in upfront and salvage regimens. A trend of improved survival was observed for those who had class switch when comparing the novel agents in their upfront and salvage regimens, although it was not statistically significant due to the limited number of patients in each subgroup (Figure S3). 

### 3.3. Survival Outcomes after First Relapse 

**Figure 1:**
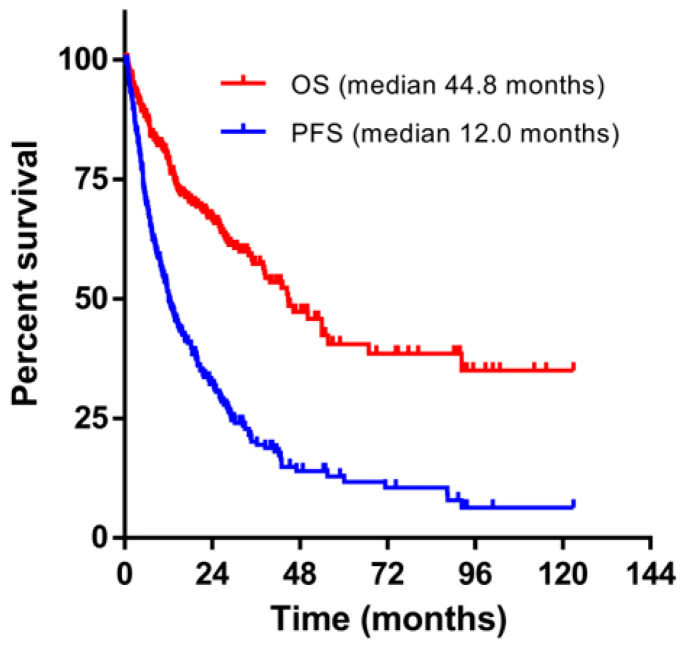
Overall survival (OS) and progression free survival (PFS) of all patients with first relapsed myeloma (n = 300).

The median PFS and OS of all patients after first relapse was 12.0 and 44.8 months, respectively (Figure 1). 

In the testing cohort, variables impacting PFS and OS were first selected from univariate analysis (Table 2). As expected, a deeper salvage response (i.e., ≥VGPR) was associated with superior PFS (hazard ratio 0.447, *p* < 0.001) and OS (hazard ratio 0.460, *p* = 0.017). However, independent prognostic factors before knowing the response are important clinically and could help to make therapeutic decisions at salvage. Relevant variables impacting PFS and OS upon multivariate analysis include non-hyperdiploid karyotype at diagnosis, clinical relapse, and treatment sequence (Table 3). Baseline ISS and high FISH were not significant after multivariate adjustment. 

**Figure 2:**
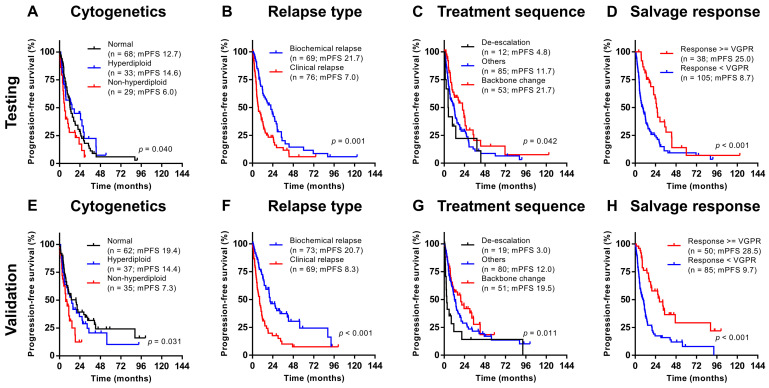
Progression free survival categorized according to different prognostic factors in the testing ((**A**) cytogenetics; (**B**) relapse type; (**C**) treatment sequence; (**D**) salvage response) and validation ((**E**) cytogenetics; (**F**) relapse type; (**G**) treatment sequence; (**H**) salvage response) cohorts.

**Figure 3:**
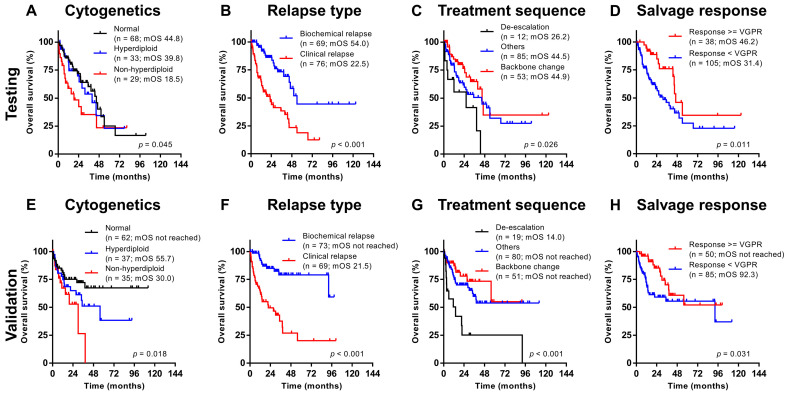
Overall survival categorized according to different prognostic factors in the testing ((**A**) cytogenetics; (**B**) relapse type; (**C**) treatment sequence; (**D**) salvage response) and validation ((**E**) cytogenetics; (**F**) relapse type; (**G**) treatment sequence; (**H**) salvage response) cohorts.

In the validation cohort, all three prognostic factors could categorize patients into subgroups with significantly different PFS and OS, consistent with the observations in the testing cohort (Figure 2 and Figure 3). Their significance remains after incorporating salvage response into the multivariate analysis (Table S5). 

## 4. Discussion

Although MM remains incurable, novel agents have drastically changed the treatment paradigms and offered various possibilities. The choice of treatment at first relapse is critical, as subsequent remissions are typically shorter [6]. However, the optimal treatment sequence has not been well defined. Recent randomized trials showed the superiority of triplet with two novel agents in the relapsed setting, with a deeper response, higher remission rate, and longer PFS [7,8,9,10,11,12]. Of note, the unprecedented hazard ratios of PFS achieved by adding daratumumab triggered great enthusiasm to consider a daratumumab-based regimen as the preferred choice at first relapse [11,12]. However, the decision always needs to be adapted to not only the patient’s characteristics, but costs and availabilities of novel agents. Therefore, it is particularly interesting to study the natural history of first relapsed MM patients in a real-world setting, which could show the salvage treatment chosen during daily practice and hopefully help to identify prognostic factors determining outcomes after relapse. 

Our study described clinical features, salvage treatment, and survival outcomes of first relapsed MM patients seen between 2004 and 2019 from two tertiary institutes in Singapore. It is informative to compare the findings of our study with a previous similar study that included relapsed MM patients between 1985 and 1998, the period before novel agents were available [6]. The study showed the median response duration of initial and salvage treatment as 9.9 and 7.3 months, respectively, which were significantly shorter than our observation. In addition, the median OS from salvage was 17.1 months, as compared with 44.8 months in our current cohort. Both findings are most probably due to increasing access to novel agents during the period of our study, which is also reflected by using newer agents during salvage compared to initial treatment in our cohort. The use of these agents in not only first but subsequent relapses may also explain the significant difference between median PFS and OS in our cohort, implying a chance of successful salvage even after two lines of therapy. 

It is important to understand the prognostic factors for MM patients at first relapse, which could guide therapeutic decisions at salvage. Most data on well-known prognostic factors, including a patient’s characteristics (e.g., age and functional status), staging, and disease features (e.g., metaphase cytogenetics and FISH), are from the time of diagnosis [20]. Their particular values at relapse are not fully clear but are often used to identify high-risk patients in trials. In addition, more parameters, especially initial treatment and features at first relapse, also deserve careful consideration. The three prognostic factors that affect survival after first relapse identified in our study have their unique clinical relevance. Non-hyperdiploid karyotype at diagnosis may identify a more proliferative underlying myeloma clone and has recently demonstrated its consistency with FISH to predict high-risk patients at diagnosis [21]. Although not informative in the majority of patients, in those with abnormal non-hyperdiploid karyotype, this clearly identifies a subgroup of myeloma with aggressive biology that is associated with difficult salvage and poor post-relapse survival, even in the modern era with novel therapies [22,23]. In addition, type of relapse also reflects the aggressiveness of disease, and clinical relapse correlates with inferior outcome [24]. So far, there is no clear consensus regarding the best regimen at first relapse; therefore, treatment sequence is an area of interest to explore. Although the current study is not designed to demonstrate the significance of a particular sequence, our result is consistent with the notion that at least one novel agent that the patient was never exposed to previously should be used, either by switching the therapeutic backbone or incorporating it directly, which is supported by the idea that initial treatment provides a selective pressure for resistant clones underlying disease relapse [25,26,27].

It is also interesting to note that ISS, LDH, and high-risk FISH, three traditional prognostic factors at diagnosis, are not significant for post-first relapse survival. These prognostic factors are derived and established in large datasets of newly diagnosed patients; however, the role of baseline ISS, LDH, and FISH in post-relapse outcome has not been systematically studied. Despite this, they are always used as variables to define high-risk disease in the relapse setting. Our study suggests that there may be other more important factors in determining post-relapse survival. One important caveat is that our cut-off for 17p13 del is 10%. Studies have shown that it is those with a high clonal fraction of 17p13 deletion that have poorer prognosis [28]. This may have affected the prognostic impact of high-risk FISH in our cohort. However, due to the low number, this is unlikely to have a statistically significant impact. 

Salvage response was not incorporated into our initial multivariate analysis, as (1) we would like to explore the factors impacting outcome that could help therapeutic decision making before commencement of salvage and (2) there is a well-known relationship between the depth of response and outcome in the relapsed setting [29,30,31]. As expected, there is a significant association between the salvage response and survival. 

Our study has its limitations due to its retrospective nature. First, assessment at relapse is not fully standardized and metaphase cytogenetics as well as FISH are not available for most cases. Therefore, we cannot make any conclusions about the usefulness of FISH or metaphase cytogenetics at relapse. Second, the prognostic value of a specific salvage regimen or sequence is not assessable in view of limited cases of each therapy. In addition, the impact of lenalidomide maintenance is not evaluated, as the study period spans over 15 years, a time when major changes in lenalidomide use, its accessibility, and our local government reimbursement have occurred. 

## 5. Conclusions

In conclusion, our study provides a real-world landscape of first relapsed myeloma and highlights the prognostic importance of karyotype at diagnosis, relapse type, and treatment sequence. Access to newer agents is likely to further improve the outcome of relapsed patients. How to justify their use in the real-world practice with relatively limited resources needs to be addressed carefully in further studies. 

## Figures and Tables

**Table 1:** Patients’ characteristics.

Variables n (%) or Median (range) at Diagnosis	All Patients (n = 300)	Testing Cohort (n = 150)	Validation Cohort (n = 150)	*p*-Values
Male	166 (55.3)	79 (52.6)	87 (58.0)	0.353
Heavy chain type IgG/IgA/others	181/52/30 (n = 263)	86/24/16 (n = 126)	95/28/14 (n = 137)	0.807
Anemia *	188 (63.9) (n = 294)	93 (62.8) (n = 148)	95 (65.1) (n = 146)	0.690
Hypercalcemia	40 (14.4) (n = 277)	20 (14.3) (n = 140)	20 (14.6) (n = 137)	0.941
Renal insufficiency	66 (22.7) (n = 291)	36 (24.3) (n = 148)	30 (21.0) (n = 143)	0.496
Bone lesions	187 (66.5) (n = 281)	102 (73.9) (n = 138)	85 (59.4) (n = 143)	0.010
Extramedullary plasmacytoma	54 (19.2) (n = 281)	31 (22.5) (n = 138)	23 (16.1) (n = 143)	0.175
Elevated LDH	45 (19.6) (n = 230)	19 (17.1) (n = 111)	26 (21.8) (n = 119)	0.366
ISS I/II/III	44/104/132 (n = 280)	24/49/70 (n = 143)	20/55/62 (n = 137)	0.700
Cytogenetics normal/hyperdiploid/non-hyperdiploid **	130/70/64 (n = 264)	68/33/29 (n = 130)	62/37/35 (n = 134)	0.604
High-risk FISH	76 (31.8) (n = 239)	37 (29.8) (n = 126)	39 (34.5) (n = 113)	0.394
t(4;14)/t(14;16)/del(17p) ***	43/2/42	20/1/19	23/1/23	0.986
**Upfront Treatment**				
ASCT	124 (41.3)	58 (38.7)	66 (44.0)	0.348
1st line regimen: novel agents	284 (94.7)	141 (94.0)	143 (95.3)	0.607
Best response ≥ VGPR	141 (48.6) (n = 290)	72 (49.3) (n = 146)	69 (47.9) (n = 144)	0.812
**At Relapse**				
Age (years)	66 (36–94)	66.5 (42–91)	65 (36–94)	0.078
Time from diagnosis to 1st relapse (months)	22.7 (1.1–97.0)	23.0 (2.6–76.4)	21.5 (1.1–97.0)	0.288
Anemia	85 (29.8) (n = 285)	41 (28.4) (n = 144)	44 (31.2) (n = 141)	0.614
Hypercalcemia	13 (5.5) (n = 235)	6 (5.0) (n = 121)	7 (6.1) (n = 114)	0.692
Renal insufficiency	40 (14.1) (n = 283)	20 (14.0) (n = 143)	20 (14.3) (n = 140)	0.942
Bone lesions	102 (68.0) (n = 150)	55 (71.4) (n = 77)	47 (64.4) (n = 73)	0.355
Extramedullary plasmacytoma	43 (21.3) (n = 202)	24 (25.0) (n = 96)	19 (17.9) (n = 106)	0.220
Clinical relapse	145 (50.5) (n = 287)	76 (52.4) (n = 145)	69 (48.6) (n = 142)	0.517
**Salvage treatment**				
ASCT	17 (5.7)	5 (3.3)	12 (8.0)	0.080
2nd line regimen: newer agents	57 (19.0)	29 (19.3)	28 (18.6)	0.883
Treatment sequence: backbone change	104 (34.7)	53 (35.3)	51 (34.0)	0.808
Best response ≥ VGPR	88 (31.7) (n = 278)	38 (26.6) (n = 143)	50 (37.0) (n = 135)	0.061

Abbreviations: LDH, lactate dehydrogenase; ISS, international staging system; FISH, fluorescence in-situ hybridization; VGPR, very good partial response; ASCT, autologous stem cell transplantation. * End organ damages, including anemia, hypercalcemia, renal insufficiency, and bone lesions, are defined according to International Myeloma Working Group consensus 2014. ** Non-hyperdiploid metaphase karyotype includes hypodiploid, pseudodiploid, and near-tetraploid karyotypes. *** The high-risk features are not mutually exclusive.

**Table 2:** Univariate Cox analysis of the testing cohort.

Variables at Diagnosis	OS HR (95% CI)	*p*-Values	PFS HR (95% CI)	*p*-Values
Male	1.478 (0.889–2.457)	0.132	0.951 (0.655–1.380)	0.791
Heavy chain type IgG	1.000		1.000	
IgA	0.803 (0.371–1.736)	0.577	1.150 (0.696–1.900)	0.587
Others	0.966 (0.342–2.728)	0.948	1.161 (0.555–2.431)	0.692
Anemia	1.348 (0.789–2.302)	0.274	1.534 (1.020–2.307)	0.040
Hypercalcemia	1.169 (0.574–2.380)	0.667	1.281 (0.761–2.158)	0.351
Renal insufficiency	1.123 (0.640–1.970)	0.685	0.819 (0.532–1.262)	0.365
Bone lesions	1.233 (0.676–2.250)	0.494	1.401 (0.878–2.236)	0.157
Extramedullary plasmacytoma	1.561 (0.920–2.649)	0.099	1.161 (0.742–1.817)	0.513
Elevated LDH	1.131 (0.500–2.558)	0.767	1.165 (0.665–2.042)	0.593
ISS I	1.000		1.000	
II	2.147 (0.812–5.675)	0.123	1.464 (0.784–2.733)	0.232
III	2.612 (1.017–6.706)	0.046	1.654 (0.908–3.012)	0.100
Cytogenetics normal	1.000		1.000	
hyperdiploid	1.137 (0.596–2.170)	0.697	0.892 (0.542–1.468)	0.653
non-hyperdiploid	1.945 (1.045–3.619)	0.036	1.715 (1.053–2.794)	0.030
High-risk FISH	1.588 (0.921–2.737)	0.096	1.297 (0.835–2.015)	0.247
**Upfront Treatment**				
ASCT	0.907 (0.534–1.541)	0.718	0.938 (0.630–1.397)	0.753
1st line regimen: novel agents	1.165 (0.421–3.227)	0.769	1.811 (0.788–4.162)	0.162
Best response ≥ VGPR	1.340 (0.806–2.230)	0.259	0.851 (0.582–1.245)	0.406
**At Relapse**				
Age > median	1.307 (0.788–2.166)	0.300	1.055 (0.724–1.536)	0.781
Time to relapse > median	0.617 (0.373–1.020)	0.060	0.465 (0.317–0.681)	<0.001
Relapse after Jan 2016 *	1.310 (0.741–2.317)	0.353	1.064 (0.707–1.600)	0.767
Anemia **	2.842 (1.667–4.846)	<0.001	2.182 (1.440–3.307)	<0.001
Hypercalcemia	2.397 (0.734–7.829)	0.148	2.307 (0.923–5.767)	0.074
Renal insufficiency	1.193 (0.586–2.426)	0.627	0.767 (0.437–1.347)	0.356
Bone lesions	2.341 (1.066–5.145)	0.034	1.675 (0.925–3.033)	0.089
Extramedullary plasmacytoma	1.865 (0.910–3.821)	0.089	2.179 (1.285–3.694)	0.004
Clinical relapse	3.364 (1.931–5.862)	<0.001	1.896 (1.289–2.789)	0.001
**Salvage treatment**				
ASCT	0.411 (0.057–2.975)	0.379	0.337 (0.083–1.369)	0.128
2nd line regimen: newer agents	0.656 (0.323–1.334)	0.244	0.686 (0.403–1.167)	0.165
Treatment sequence: de-escalation	1.000		1.000	
others	0.463 (0.213–1.005)	0.051	0.722 (0.380–1.371)	0.320
backbone change	0.321 (0.137–0.754)	0.009	0.440 (0.220–0.880)	0.020
Best response ≥ VGPR	0.460 (0.243–0.869)	0.017	0.447 (0.284–0.703)	<0.001

Abbreviations: LDH, lactate dehydrogenase; ISS, international staging system; FISH, fluorescence in-situ hybridization; VGPR, very good partial response; ASCT, autologous stem cell transplantation; OS, overall survival; PFS, progression free survival; HR, hazards ratio; CI, confidence interval. * The date was chosen as newer agents (e.g., carfilzomib, daratumumab) were more widely used after that in Singapore. ** Because anemia at relapse and clinical relapse were highly correlated (*p* = 0.005), the latter variable was used in multivariate analysis due to its clinical significance.

**Table 3:** Multivariate Cox analysis of the testing cohort.

Variables at Diagnosis	OS HR (95% CI)	*p*-Values	PFS HR (95% CI)	*p*-Values
Cytogenetics: normal	1.000		1.000	
hyperdiploid	1.232 (0.630–2.408)	0.543	1.670 (0.820–3.402)	0.158
non-hyperdiploid	2.028 (1.026–4.008)	0.042	2.386 (1.203–4.732)	0.013
**At relapse**				
Clinical relapse	3.431 (1.929–6.103)	<0.001	5.351 (2.826–10.133)	<0.001
**Salvage treatment**				
Treatment sequence: de-escalation	1.000		1.000	
others	0.393 (0.179–0.861)	0.020	0.336 (0.151–0.748)	0.008
backbone change	0.387 (0.160–0.939)	0.036	0.319 (0.127–0.806)	0.016

Abbreviations: OS, overall survival; PFS, progression free survival; HR, hazard ratio; CI, confidence interval.

## Supplementary Materials

The following are available online at https://www.mdpi.com/2072-6694/12/7/1759/s1, Figure S1: Salvage responses according to treatment regimens. Thal, thalidomide and Len, lenalidomide, Figure S2: Progression free survival of all patients categorized according to treatment sequence with similar survival trends. Figure S3: Progression free survival categorized according to upfront (A, bortezomib; B, immunomodulatory drugs (IMiDs); C, bortezomib + IMiDs) and salvage regimens. Table S1: Initial treatment regimens. Table S2: Salvage treatment regimens. Table S3: Depth of response. Table S4: Progression free survival (PFS) of patients with different treatment sequences. Table S5: Multivariate Cox analysis of the testing cohort (including salvage response). Method. Interphase fluorescence in-situ hybridization (FISH) of multiple myeloma samples.
