# Supplementary material for: Natural History and Prognostic Factors at First Relapse in Multiple Myeloma
Source: Cancers (Basel). 2020 Jul 2;12(7):1759. doi: 10.3390/cancers12071759 (PMC7409309; doi:10.3390/cancers12071759)
Supplement: Supplementary file 1 [file cancers-12-01759-s001.zip › Supplementary method (2020-6-23).docx]

Supplementary Method

Interphase fluorescence in-situ hybridization (FISH) of multiple myeloma samples

Bone marrow aspirate samples of multiple myeloma patients enriched for nucleated cells with cytoplasmic light chain staining were used for FISH analysis before 2018. After that, CD138 immunomagnetic beads-mediated sorting has been introduced to enrich plasma cells from bone marrow aspirates. We evaluate 200 cells for whole bone marrow samples, or 100 cells for CD138 sorted samples. We established the cutoff values as mean +3 standard deviation from normal control samples, and their values are re-validated every year to ensure there is no significant deviation from yearly samples and probe lots. In general, the cut-off is 10% for different probes, including 17p13 deletion.
