# Supplementary material for: Natural History and Prognostic Factors at First Relapse in Multiple Myeloma
Source: Cancers (Basel). 2020 Jul 2;12(7):1759. doi: 10.3390/cancers12071759 (PMC7409309; doi:10.3390/cancers12071759)
Supplement: Supplementary file 1 [file cancers-12-01759-s001.zip › Supplementary tables.docx]

**Table S1.** Initial treatment regimens.

| **Initial treatment** | **n = 300 (%)** |
| --- | --- |
| Conventional therapy | 16 (5.3) |
| Novel agent-based: bortezomib | 135 (45.0) |
| thalidomide | 65 (21.7) |
| lenalidomide | 2 (0.7) |
| bortezomib + thalidomide | 61 (20.3) |
| bortezomib + lenalidomide | 11 (3.7) |
| Newer agent-containing: carfilzomib | 10 (3.3) |

**Table S2.** Salvage treatment regimens.

| **Salvage regimen** | **n = 300 (%)** |
| --- | --- |
| Conventional therapy | 31 (10.3) |
| Novel agent-based: bortezomib | 44 (14.7) |
| thalidomide | 38 (12.7) |
| lenalidomide | 73 (24.3) |
| bortezomib + thalidomide | 21 (7.0) |
| bortezomib + lenalidomide | 36 (12.0) |
| Newer agent-containing: carfilzomib | 21 (7.0) |
| ixazomib | 16 (5.3) |
| pomalidomide | 7 (2.3) |
| daratumumab* | 11 (3.7) |
| dinaciclib | 2 (0.7) |

* Daratumumab was used in combination therapy (i.e., daratumumab/thalidomide/dexamethasone n = 2; daratumumab/lenalidomide/dexamethasone n = 7; daratumumab/bortezomib/dexamethasone n = 2).

**Table S3.** Depth of response.

| **Response category** | **Upfront (n = 290)** | **Salvage (n = 278)** |
| --- | --- | --- |
| Complete response (CR) | 57 (19.7) | 39 (14.0) |
| Very good partial response (VGPR) | 84 (29.0) | 49 (17.6) |
| Partial response (PR) | 122 (42.1) | 99 (35.6) |
| Minimal response (MR) | 5 (1.7) | 9 (3.2) |
| Stable disease (SD) | 17 (5.9) | 46 (16.5) |
| Progressive disease (PD) | 5 (1.7) | 36 (12.9) |

**Table S4.** Progression free survival (PFS) of patients with different treatment sequences.

| **Treatment sequence No.** | **Treatment sequence description** | **Patient No.** | **Median PFS (months)*** |
| --- | --- | --- | --- |
| 1 | Conventional therapy as salvage, regardless of initial treatment | 31 | 4.6 |
| 2 | Same novel agent as backbone | 55 | 11.7 |
| 3 | Generation escalation of immunomodulatory drug (e.g., thalidomide escalated to lenalidomide) | 27 | 13.0 |
| 4 | Generation escalation of proteasome inhibitor (e.g., bortezomib escalated to carfilzomib) | 25 | 12.7 |
| 5 | Backbone change: immunomodulatory drug to proteasome inhibitor | 21 | 34.5 |
| 6 | Background change: proteasome inhibitor to immunomodulatory drug | 72 | 19.5 |
| 7 | Daratumumab based salvage, regardless of initial treatment | 11 | 19.3 |
| 8 | Others (e.g., adding on a different class of novel agent, for example from bortezomib-dexamethasone to bortezomib-lenalidomide-dexamethasone) | 58 | 10.8 |

* *p* value by log-rank test: 0.012.

** treatment sequences were further categorized into three subgroups, namely de-escalation (1), backbone change (5, 6 and 7) and others (2, 3, 4 and 8), according to their PFS trends.

**Table S5.** Multivariate Cox analysis of the testing cohort (including salvage response).

| **Variables** | **OS** |  | **PFS** |  |
| --- | --- | --- | --- | --- |
|  | **HR (95% CI)** | ***P* values** | **HR (95% CI)** | ***P* values** |
| ***At diagnosis*** |  |  |  |  |
| Cytogenetics: normal | 1.000 |  | 1.000 |  |
| hyperdiploid | 1.385 (0.711-2.699) | 0.338 | 1.221 (0.715-2.088) | 0.464 |
| non-hyperdiploid | 2.333 (1.190-4.574) | 0.014 | 1.835 (1.093-3.078) | 0.022 |
| ***At relapse*** |  |  |  |  |
| Clinical relapse | 3.725 (2.089-6.642) | < 0.001 | 1.594 (1.015-2.504) | 0.043 |
| ***Salvage treatment*** |  |  |  |  |
| Treatment sequence: de-escalation | 1.000 |  | 1.000 |  |
| others | 0.347 (0.157-0.768) | 0.009 | 0.647 (0.334-1.254) | 0.197 |
| backbone change | 0.412 (0.170-0.996) | 0.049 | 0.491 (0.236-1.025) | 0.058 |
| Best response >= VGPR | 0.353 (0.175-0.710) | 0.004 | 0.380 (0.231-0.623) | < 0.001 |

Abbreviations: OS, overall survival; PFS, progression free survival; HR, hazards ratio; CI, confidence interval; VGPR, very good partial response.
